# DAIR in treating chronic PJI after total knee arthroplasty using continuous local antibiotic perfusion therapy: a case series study

**DOI:** 10.1186/s12891-024-07165-y

**Published:** 2024-01-05

**Authors:** Yukichi Zenke, Yasuhito Motojima, Kohei Ando, Kenji Kosugi, Daishi Hamada, Yasuaki Okada, Naohito Sato, Daichi Shinohara, Hitoshi Suzuki, Makoto Kawasaki, Akinori Sakai

**Affiliations:** 1 Department of Trauma Reconstruction, University of Occupational and Environmental Health, 1-1 Iseigaoka Yahatanisiku, Kitakyusyu City Fukuoka Prefecture, Japan; 2 Department of Orthopaedic Surgery, University of Occupational and Environmental Health, Kitakyusyu City Fukuoka Prefecture, Japan; 3 Department of Arthroplasty Center, University of Occupational and Environmental Health, Kitakyusyu City Fukuoka Prefecture, Japan

**Keywords:** Continuous local antibiotic perfusion therapy (CLAP), Chronic Infection, Postoperative total knee arthroplasty, Debridement, Antibiotics, Implant retention (DAIR)

## Abstract

**Background:**

Antimicrobial agents are administered via intramedullary antibiotic perfusion (iMAP)/intrasoft tissue antibiotic perfusion (iSAP) to infected lesions to control osteoarticular and soft tissue infections. Continuous local antibiotic perfusion (CLAP) has been reported to be useful. This study aimed to investigate the outcomes of DAIR combined with CLAP for chronic PJI after total knee arthroplasty performed at our hospital.

**Subjects and methods:**

Six patients (male; one case, female; five cases, mean age 79.5 years (70–94)) underwent CLAP for chronic PJI after TKA at our hospital between July 2020 and June 2022. They were followable for at least one year after surgery. Seven months (17–219), with a mean follow-up of 24.3 months (12–36). In addition to direct debridement and insert exchange, systemic antimicrobial treatment, and CLAP with gentamicin were performed using NPWT. We investigated the organisms causing the inflammation, the duration of iMAP/iSAP implantation, the maximum daily dose of GM, the maximum GM blood concentration, and the presence or absence of GM-induced adverse events.

**Result:**

Two of six patients had a recurrence of infection at five weeks and five months after initial CLAP and required repeat CLAP treatment, but all patients could preserve their components. The organisms responsible for the flare-ups were MSSA in three cases: ESBL-producing *E. coli*, mixed MSSA and streptococcal infection, *Klebsiella* pneumonia in one case each, and unknown pathogens in one case. CLAP therapy for all patients was administered eight times in 6 cases: iMAP, mean: 10.0 days (5–16); iSAP, mean: 19.3 days (15–28); GM dose, mean: 162.5 mg/day (80–240); and GM blood concentration, mean: 1.4 µg/mL (0.2-5.0). Adverse events included one case of reversible acute kidney injury during CLAP in a patient with recurrent infection.

**Summary:**

DAIR with CLAP for chronic post-TKA infection can be a useful treatment option to preserve components and allow the infection to subside, provided the implant is not markedly loosened.

**Supplementary Information:**

The online version contains supplementary material available at 10.1186/s12891-024-07165-y.

## Background

Periprosthetic joint infection (PJI) is a severe complication. According to the Second International Consensus Meeting (ICM) on Musculoskeletal Infection in 2018 [1], debridement, antibiotics, and implant retention (DAIR) with the exchange of modular components can be effective in patients with PJI. The best advantage in performing DAIR of the prosthesis is seen in early postoperative PJI and acute hematogenous PJI [2, 3], defined as symptoms existing for no longer than four weeks and if the implant is stable. Therefore, some cases need exchange arthroplasty, arthrodesis, and leg amputation because of failure of infection control [4].

**Fig. 1:**
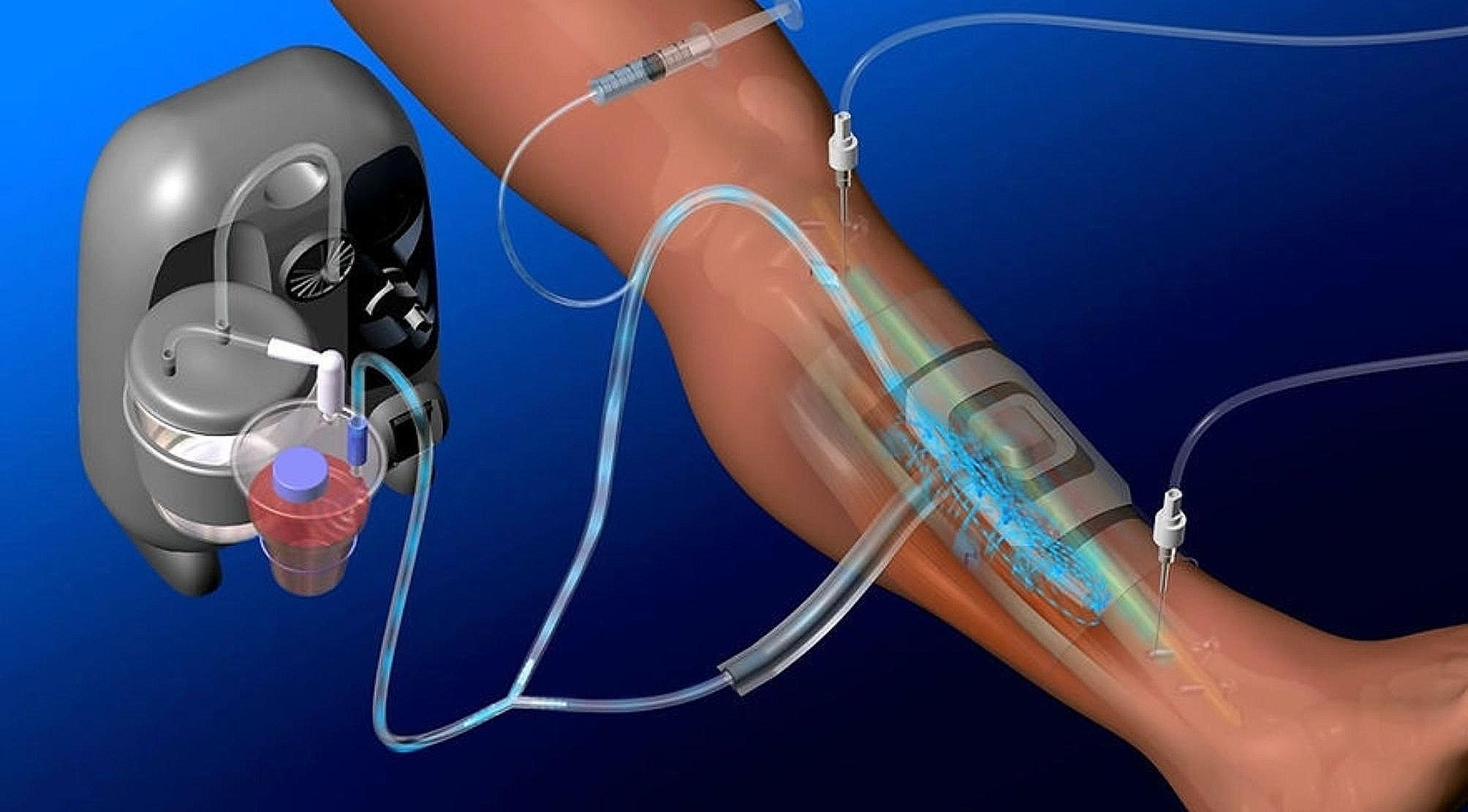
Schematic diagram of CLAP therapy for FRI after open fracture of the lower leg. https://www.ismap-clap.com

Recently, a novel treatment strategy using continuous local antibiotic perfusion (CLAP) for treating bone and soft tissue infections has been reported [5–8]. CLAP therapy is considered an innovative system that can maintain a high concentration of antibiotics locally at the infected site for a long duration. In the treatment, antibiotics are continuously infused using syringe pumps via dual-lumen tubes (Salam samp tube®: Nihon Covidien Co.) (Appendix Fig. A), intrasoft-tissue antibiotic perfusion (iSAP), and/or via bone marrow needles (iMAP needle®: Cubex Medical Co.) (Appendix Fig. B), intramedullary antibiotic perfusion (iMAP), to deliver highly concentrated antibiotics to the target sites. In particular, the intrajoint antibiotic perfusion (iJAP) technique is employed. We have already utilized CLAP therapy to treat fracture-related infections (FRI) and have suggested its validity of it (Fig. 1) [7]. Thus, we believed this therapy could be a powerful tool to eradicate infection even in cases of PJI; we have utilized this therapy to treat infection after total knee arthroplasty (TKA). We experienced a case of PJI in an elderly patient with suspected PJI in the relatively early postoperative period and a poor general condition despite chronic infection, in which CLAP was successfully performed to preserve the prosthesis and control infection. In the present study, we investigated the treatment outcomes of DAIR with CLAP therapy for patients with chronic infection after TKA.

## Methods

This case series study includes six patients who received DAIR with CLAP therapy for late-phase infection after TKA at the University of Occupational and Environmental Health, Japan Hospital, for at least 12 months between July 2020 and June 2022.

There was one male and five female patients, with a mean age of 79.5 (70–94) years.

CLAP therapy was carried out according to the treatment protocol described in the Surgical procedure section. Briefly, we carried out DAIR (*the surfaces were replaced in all cases), placed iJAP & iMAP routes, and perfused gentamicin (GM) via these routes. The concentration of gentamicin sulfate used topically was similar in all cases, with 60 mg of GM being dissolved in 50 ml of saline (1200 µg/ml1) for each route. However, as the number of courses varied from each case, the total daily GM use averaged 162.5 mg (80–240). We retrospectively collected the following data regarding the patients: age, sex, responsible bacteria, duration of use of the iJAP route, duration of use of the iMAP route, maximum dosages of GM for CLAP, maximum blood concentration of GM (tested by postoperative day 3), adverse events, and complications.

### Diagnosis

It was examined whether the following clinical findings were present around the patients’ knees: redness, heat, swelling, fistula, and pus. And we follow the ICM diagnostic criteria. A blood test including C-reactive protein (CRP) concentration was also examined; CRP positivity was defined as over 1.0 mg/dL under the criteria [1]. Bacterial culture tests of synovial fluid, pus, and infected tissue were collected before and under DAIR. We diagnosed infection after TKA if the patients presented clinical findings, including CRP positivity and culture positivity.

### Surgical procedure

Further information on the surgical procedure is detailed in the Appendix: Surgical procedure (including Video 1 and Figure C, D).

## Result

Two of the six cases were complicated by a necrotizing soft tissue infection (NSTI) in the thigh and lower leg and by postoperative TKA infection. The mean time between TKA surgery (of these, two were after revision TKA, and four were after primary TKA and the first CLAP treatment) in all six patients was 93.7 months (17–219). A total of eight CLAP treatments were given in all six patients (two were given twice because of recurrent infection), and the infection had subsided in all patients after a mean follow-up period of 24.3 months (12–36). The organisms responsible were MSSA in three cases, *E. coli* (ESBL), *Klebsiella pneumonia*, and *Streptococcus dysgalactiae* in one case each, and unknown organisms in one case (there was a duplicate case). In addition, iJAP was performed in all cases, with an average duration of retention of 19.3 days (15–28), and iMAP was performed four times out of 8 performed in 6 patients, with an average time of retention of 10.0 days (5–16). Pre-operative culture results were negative in only one case but positive in the other five; CLAP therapy was administered in all cases from the time of the first debridement; after DAIR, all patients received antimicrobials via intravenous administration. The duration of this varies slightly from case to case but generally lasts about three weeks. Thereafter, the patients are switched to oral antimicrobials with susceptible antimicrobials, which are administered for approximately six months (as listed in Table 1).

Currently, GM is based on 60 mg per route plus 50 ml of saline (1200 µg/ml), but 40 mg or 80 mg was used in some early cases. The maximum daily dose of GM was 162.5 mg (80–240) on average. The maximum GM blood concentration, measured on postoperative day 3, averaged 1.4 µg/dL (0.2-5). Adverse events included tube removal during treatment duration in one case and renal dysfunction as a complication in one case. The overall case results are summarized in Table 1.

**Table 1:** The overall of 6 cases results

Case	Sex	Age	Causative bacteria	The time after TKA(m)	CLAP therapy	iJAP(d)	iMAP(d)	GM (mg/day)	GM max blood concentration (μ/dL)	Follow up period(m)	Intravenous antibacterial usage time(d)	Oral antibacterial usage time(m)	Results
**1**	M	84	MSSA	33	1st	17	5	120	1.5	(-)	MEPM(12d)	MINO(1w)	Recurrence (2.5M)
				(-)	2nd	19	7	160	2.1	36	CEZ(16d)	RFP,MINO(12m)	**Remission**
**2**	F	81	E. coli	125	1st	16	16	200	5	26	CMZ(35d)	CCL,MINO(3m)	**Remission**
**3**	F	94	MSSA	219	1st	15	(-)	80	0.3	12	CEZ(14d)	MINO(3m)	**Remission**
**4**	F	70	MSSA, Streptococcus dysgalactiae	75	1st	21	(-)	200	0.4	22	ABPC&CLDM(7d) CTRX(14d)	ABPC (4m)	**Remission**
**5**	F	73	Klebsiella pneumoniae	17	1st	28	(-)	120	0.5	(-)	CTRX(7d) CEZ(24d)	CCL(3m)	Recurrence (5M)
				(-)	2nd	21	12	240	1.0	32	CEZ(14d)	CCL,MINO(9m)	**Remission**
**6**	F	75	Unknown	93	1st	17	(-)	180	0.2	18	CEZ(17d)	ST, MINO(8m)	**Remission**
**Avg**		**79.5**		**93.7**		**19.3**	**10.0**	**162.5**	**1.4**	**24.3**			

## Representative case

### Patient

A 73-year-old woman, medical history: diabetes mellitus (+).

### Current medical history

**Fig. 2:**
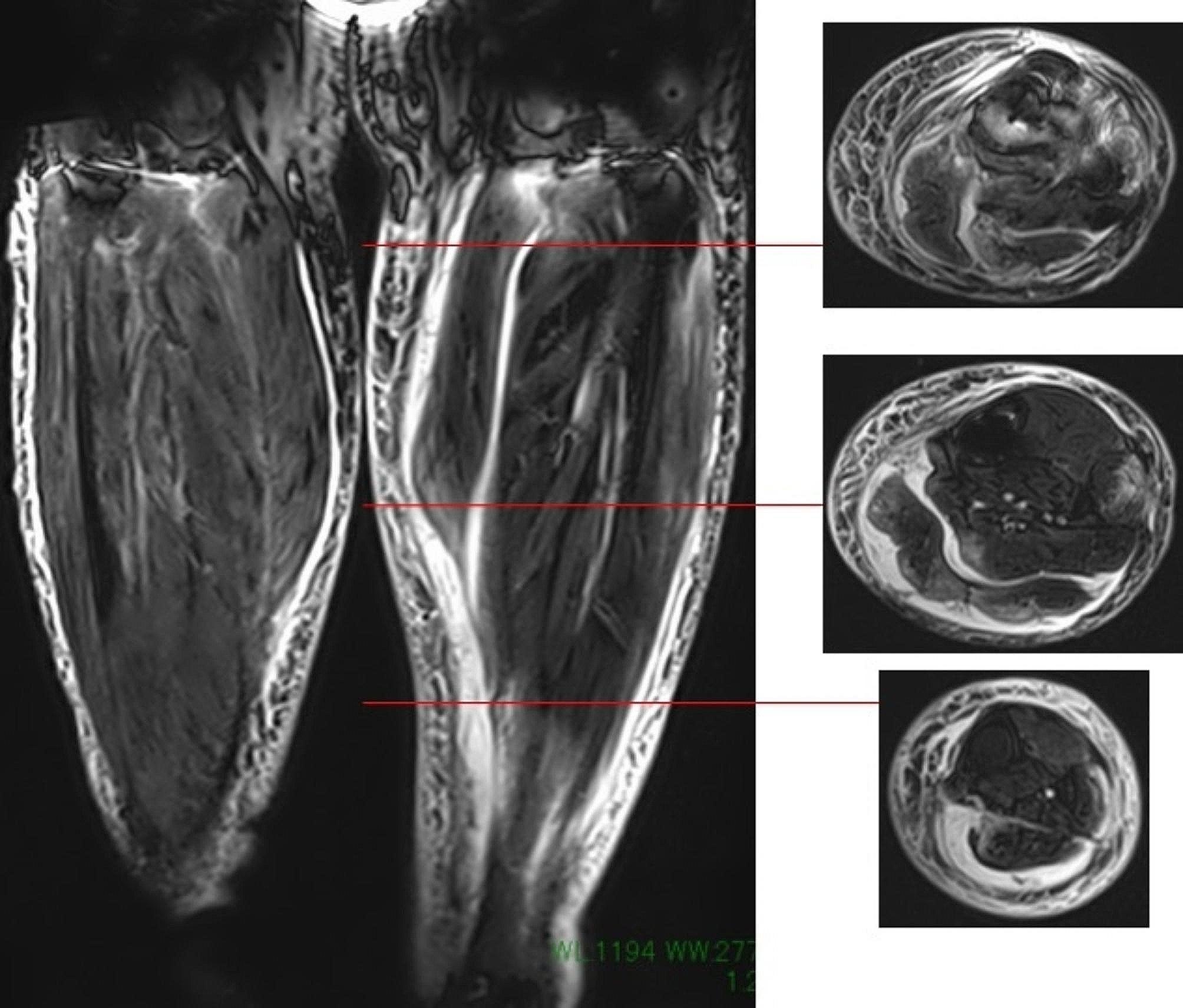
Suspicion of necrotizing soft tissue infection due to fluid accumulation around the gastrocnemius muscle on the leg MRI image

**Fig. 3:**
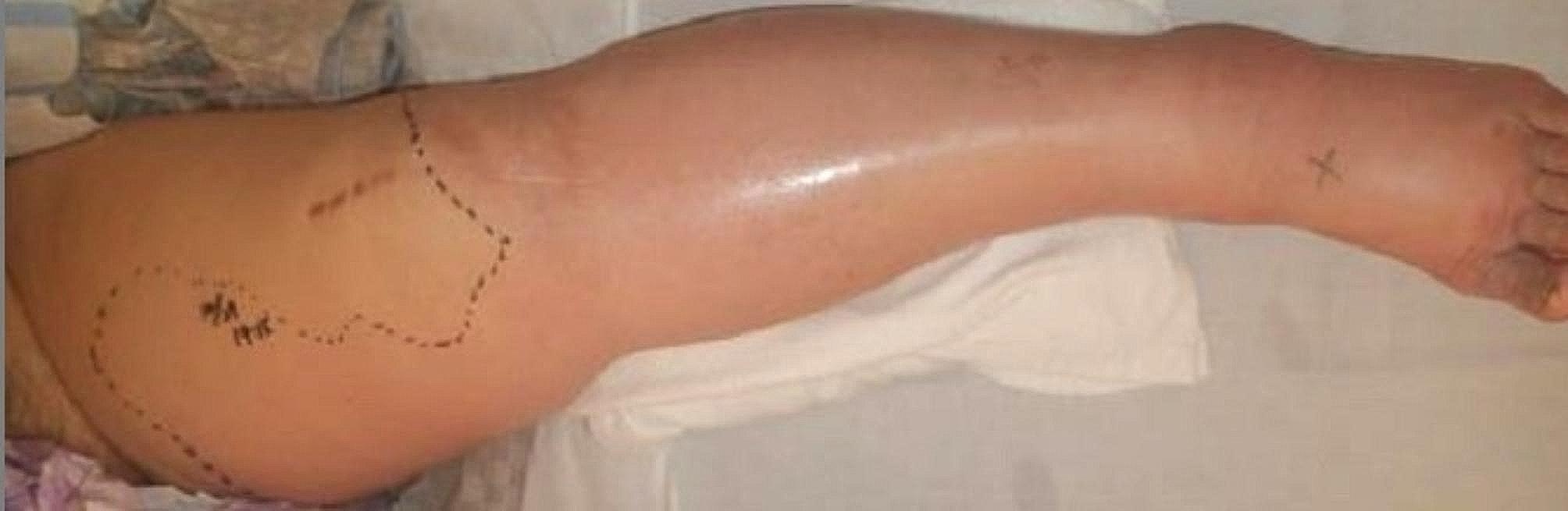
Case 1 Local findings of Lt leg at the time of first visit to our hospital the appearance of left lower limb

Left TKA (with augmentation on the tibia side) was performed by this patient’s previous doctor one year and five months ago, and she had no significant problems after the operation. Four days before her first visit to our hospital, the patient had experienced pain in her left knee joint and lower leg after walking for a long time and was being monitored. Nevertheless, the swelling and pain continued, so she visited her previous doctor the following day and was admitted to the hospital with a diagnosis of cellulitis of the left lower leg, where she was given local rest and started on intravenous antibacterial drugs (LZD). However, her symptoms did not improve. She was referred to our hospital for suspicion of necrotizing soft tissue infection due to fluid accumulation around the gastrocnemius muscle on the leg MRI image (Fig. 2) and progressive local findings. The bacterial culture of the effusion performed by the previous physician showed *Klebsiella* pneumonia. Local findings at the first visit: The patient had circumferential swelling, local heat, and redness of the lower leg (Fig. 3), and both active and passive movements of the left knee joint and ankle joint were impossible due to pain and incredibly intense tenderness around the knee joint and on the posterior aspect of the lower leg.

### Imaging findings

**Fig. 4:**
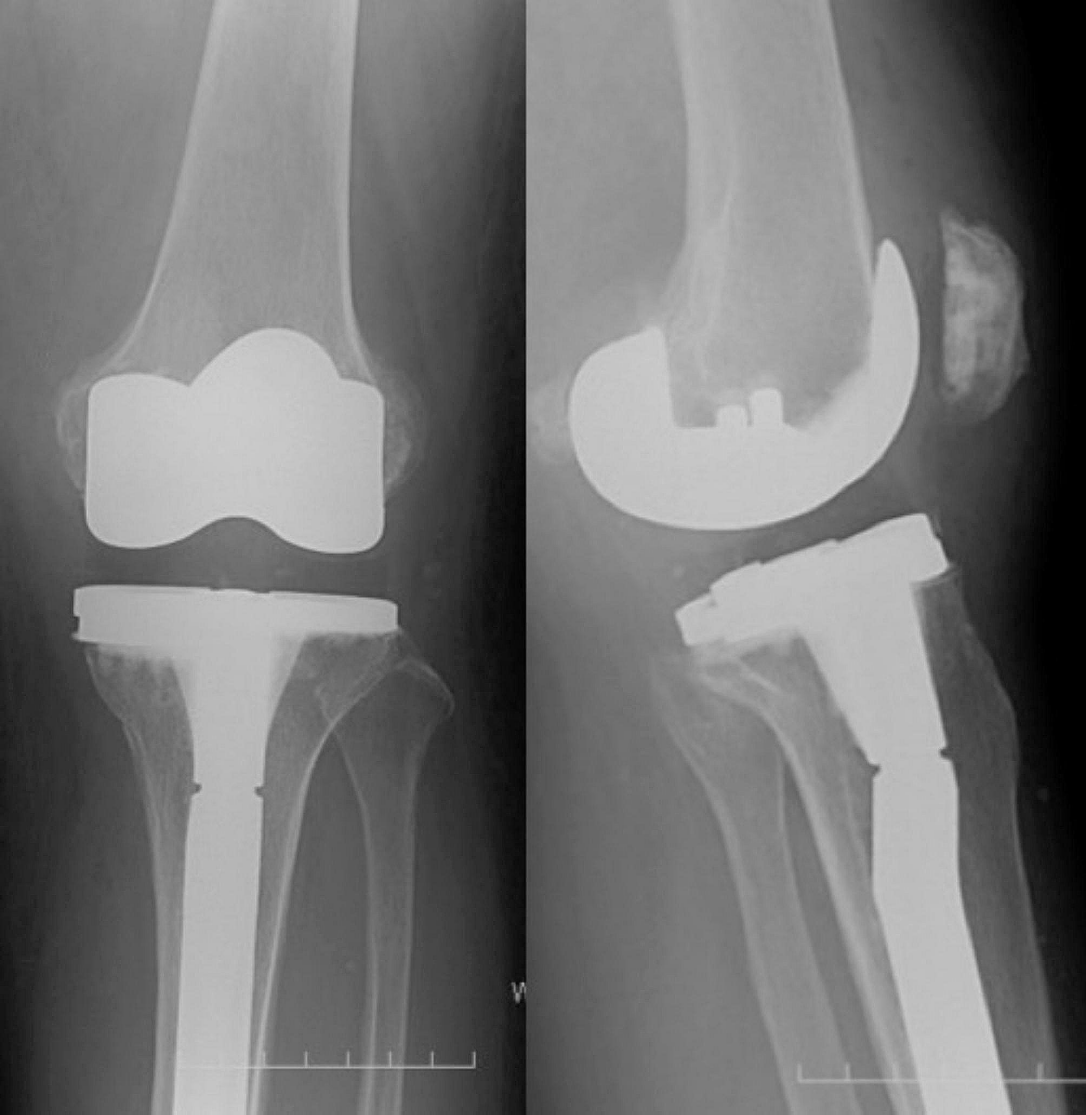
Case 1 Simple X-ray image at the time of the first visit to this hospital AP view & Lateral view

A simple X-ray of the left knee joint at the time of the patient’s first visit to our department showed no obvious implant loosening (Fig. 4).

### Blood data

Blood test results at the first visit to our department were WBC 10.0, RBC 3.65, HGB 8.9, PLT 6.7, Neutro 89.5%, TP 5.3, Alb 2.0, CK 17, and CRP 37.69.

### Intraoperative findings

**Fig. 5:**
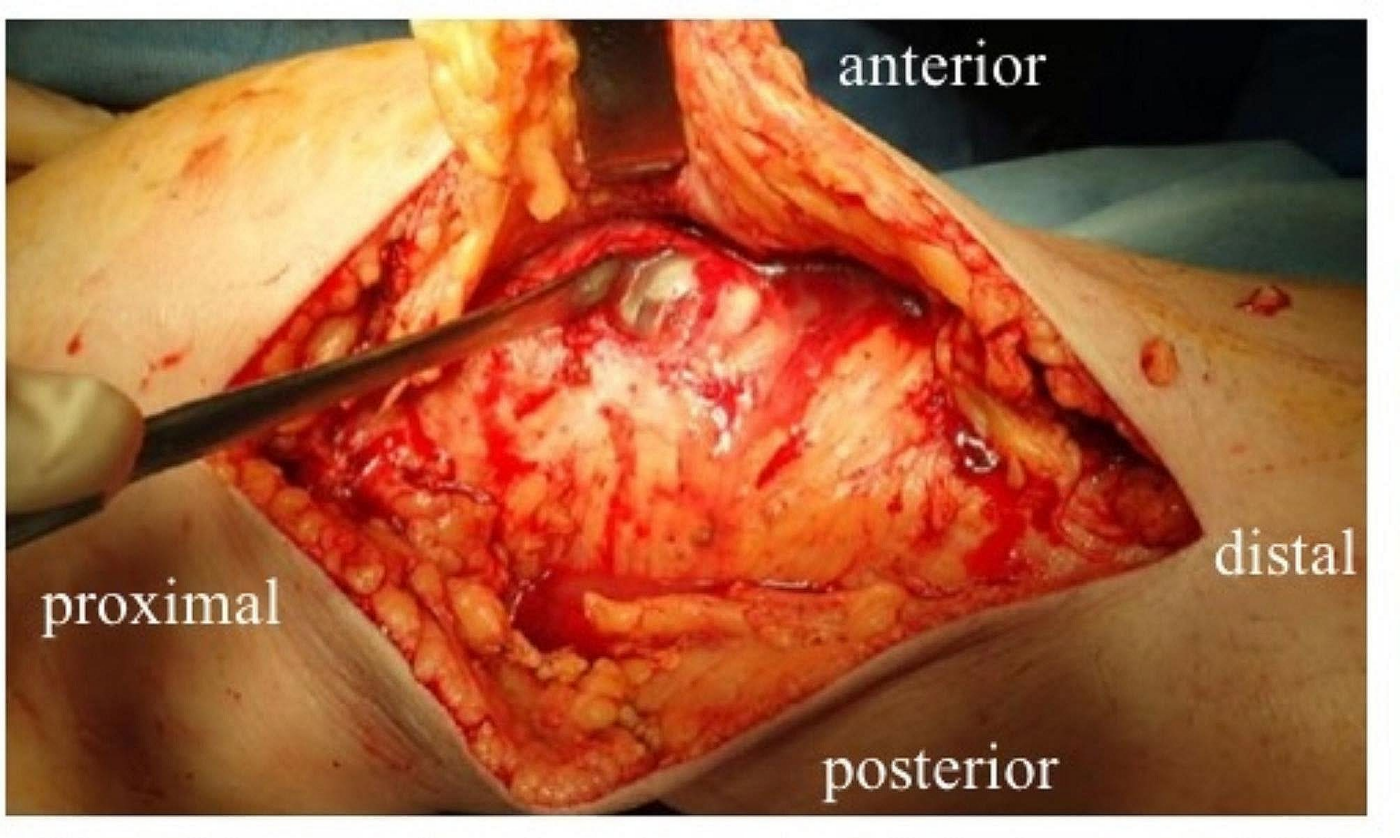
Case 1 Intraoerative findings around knee joint. Articular capsule failed and effluent drained from within the joint

**Fig. 6:**
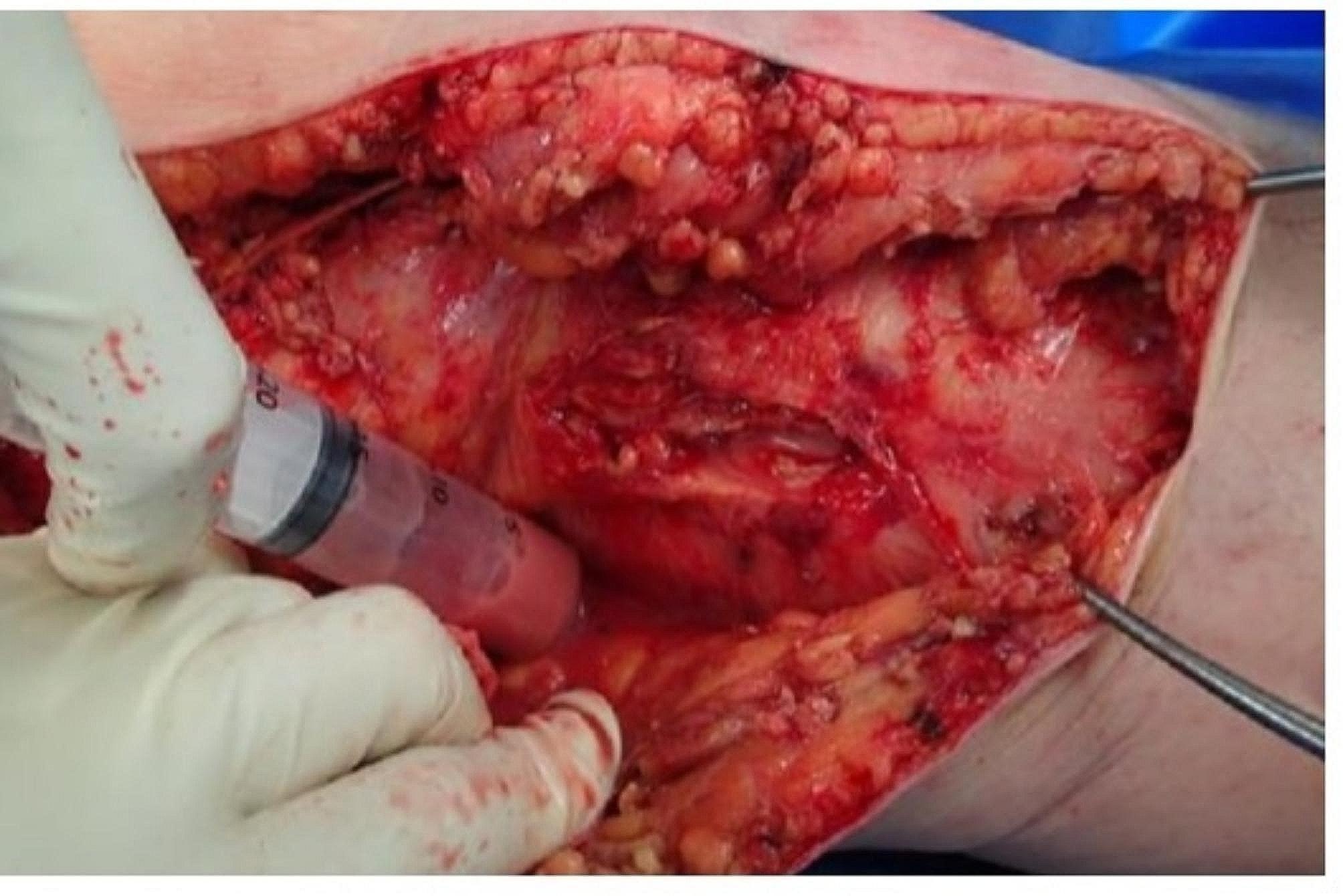
Case 1 Intraoerative findings around gastrocnemius muscle area

**Fig. 7:**
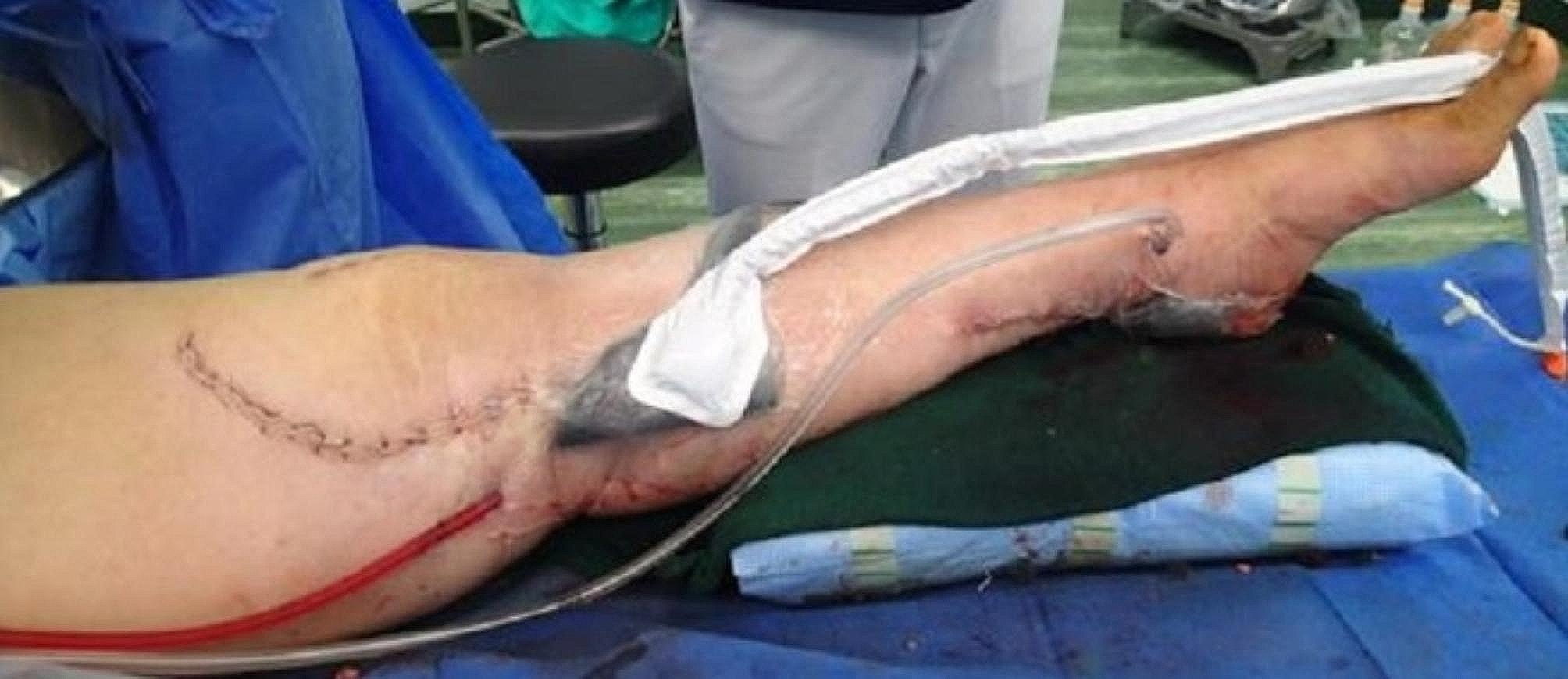
Case 1 Postoerative local findings for using CLAP therapy system

A skin incision was made around the preoperative local tender area, and internal expansion revealed that the left knee joint medial side, where the tibial augment was placed, had soft tissue failure (Fig. 5), which was trafficked into the joint, and purulent effusion was drained from the internal side. The medial articular capsule was incised to expose the intra-articular joint, the articular surface was removed, and the joint was thoroughly cleaned and debrided. The knee joint to the lower leg was also contiguous subcutaneously, with purulent effusion around the gastrocnemius muscle (Fig. 6). iJAP tubes were placed in a total of four tubes, one each in the knee joint (lateral), in the knee joint to the proximal lower leg (medial), in the distal lateral thigh, and around the Achilles tendon (Fig. 7). iMAP was not used.

### Postoperative course

**Fig. 8:**
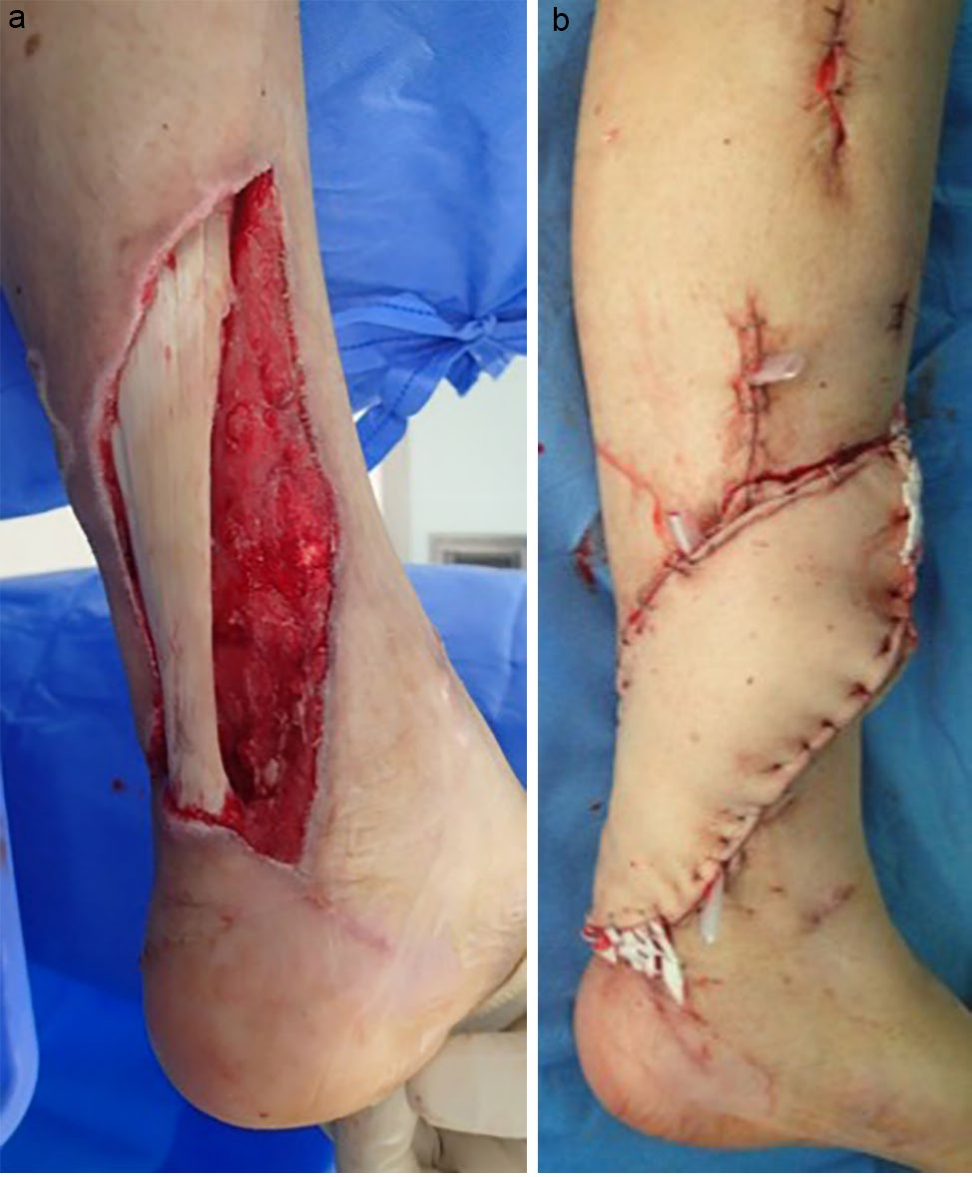
Case 1 Local findings of peri Achilles tendon soft tissue defects. Case 1 After free flap for the soft tissue defects

Immediately after surgery, the overall swelling of the left lower limb was quickly reduced, and the inflammatory response was markedly improved in the blood data. On postoperative day 7, a second look was performed as a scheduled operation. The patient underwent another round of cleaning and debridement of the intra-articular and lower leg defects, and three iJAP tubes were implanted. A further 3rd look was performed as a scheduled operation again on postoperative day 14. However, there was no intra-articular effusion, and no contamination was found; therefore, the iJAP tubes were removed. However, soft tissue necrosis was observed in the distal lower leg, which was resected, followed by soft tissue management with NPWT, and on postoperative day 28, a free anterolateral thigh flap was performed ipsilateral to the soft tissue defect in the same area (Fig. 8a and b). After that, the patient had no significant problems, but five months after the initial CLAP procedure, swelling and pain in the left knee joint appeared again. The infection flared up, but CLAP therapy was performed again (perfusion with two iJAP tubes and one iMAP pin: Video 2), which was completed after 12 days of iMAP implantation and 21 days of iJAP placement, after which the infection resolved. The patient had a good postoperative course and was transferred for rehabilitation three weeks after the last operation. At two years and eight months postoperatively, the patient remained unchanged on simple Xp and without recurrence of infection.

Another case (Case 2): See Appendix case 2.

## Discussion

PJI is one of the most frequently intractable complications of orthopedic disease. Bacteria that form biofilms around implants and in bone and soft tissue are known to have characteristics that are very different from those of the floating bacteria that would typically be identified by isolation culture. In PJI, treatment with antimicrobials, using the minimum growth arrest rate of bacteria as measured by isolation culture as an indicator, is often ineffective [9]. In the past, destroying the biofilm present in the infected foci of PJI was considered difficult. Thus, surgical removal of the infected implants or extensive debridement of the bone and soft tissue was mandatory [10]. On the other hand, some antimicrobials have been reported to be able to destroy biofilms by exposing them to high concentrations for a certain period [11]. The concentration and time required to destroy biofilms is defined as the minimum biofilm destruction concentration [12], which is reported to be 100–1000 times higher than the MIC-based concentration for the same bacteria, and MBEC has also been reported to vary with the exposure time of antimicrobials [12–14].

Surgical treatment of PJI, especially TKA postoperative infections, has thus far included the implantation of antimicrobial-containing spacers as local antimicrobial treatment after implant removal or debridement of bone and soft tissue in the case of delayed infection [15]. Polymethylmethacrylate (PMMA) and antimicrobials encapsulated within hydroxyapatite blocks (HAB) have been reported to be effective in treating residual foci of infection in the affected area by increasing local antimicrobial concentrations in the joint in a sustained release manner [15, 16]. Thus, the use of antimicrobials based on MBEC is essential in the treatment of PJI. Nevertheless, delivering MBEC-equivalent high concentrations of antimicrobials to infected lesions requires more than conventional intravenous administration, which is associated with complications.

Continuous local antibiotic perfusion: CLAP therapy is a new drug delivery system that can deliver high concentrations of antimicrobials locally [5–7]. Based on MBEC, high concentrations of antimicrobials can be delivered intramedullary (within the bone marrow). The treatment is based on MBEC and involves iMAP, iSAP, or iJAP at low flow rates to the leading site of the infection therapy. Gentamicin sulfate is used in all cases as the topically administered solution. This concentration-dependent antimicrobial agent does not induce resistance and, because of its high concentration, can also suppress resistant bacteria, further eradicating biofilms [5]. In addition, by incorporating double-lumen tubing into the NPWT device, the drug administered to the affected area can be induced by negative pressure to create a pathway to irrigate the area around the infected lesion freely [5, 17, 18]. The administered antimicrobials can also be maintained at the required concentration for the period necessary while ensuring safety by monitoring blood levels. In other words, CLAP has the potential to be a game changer in the treatment of bone and joint infections, as it allows free control of the ‘local concentration,’ ‘area of distribution,’ and ‘duration,’ which cannot be controlled by conventional local administration.

An even more beneficial and comfortable surgical procedure for the patient is DAIR, with a high degree of certainty. There is much evidence for DAIR for early infection after TKA [3, 19–21], but the treatment outcome of DAIR for late-onset infections is generally poor [19, 22–24]. We performed DAIR for chronic infection after TKA (*the average duration of infection onset since TKA surgery is 93.7 months (17–219)) using CLAP therapy [5–8, 25], for which evidence has accumulated, particularly in FRI and pyogenic arthritis. The mean postoperative follow-up period was 25.7 months (18–36), but in all six cases, the infection was quiescent. One of these patients had a flare-up of infection 2.5 months after CLAP surgery, and one had a flare-up five months after surgery but was eventually successfully treated with DAIR by readministering CLAP therapy.

The most critical point of CLAP therapy is to ensure the perfusion of high concentrations of GM to the main site of infection. Therefore, this technique requires a certain degree of experience, as a misjudgment of the main area of infection or inadequate perfusion technique (including tube placement) would mean that the benefits of CLAP may not be fully exploited. However, if the principles of this CLAP therapy are adhered to, we believe that even chronic infection after TKA surgery can be quelled without necessitating removal, provided the implant is not loosening. Further validation of this approach is needed, as there has yet to be published literature support at present, although basic experiments are currently being conducted and validated.

For example, suppose the operation is terminated with only intra-articular debridement, even though the primary site of infection is essentially in the bone marrow. In that case, the infection may not be quelled. Therefore, if osteomyelitis is strongly suspected, cleaning and debridement within the bone marrow using a bone marrow needle (Video 3) is essential. This technique can sedate the infection if performed correctly without removing the implant.

In this case series, one patient developed postoperative renal dysfunction (KDIGO classification [26] stage 3). Fortunately, the patient’s renal function normalized due to reversible changes, but as GM has been reported to cause renal dysfunction [27, 28], we measured the maximum GM blood concentration in all patients on the third postoperative day. We used a cutoff value of less than two µg/dL [29], which has been reported as a safe threshold in the past. However, because of Case 1, stricter criteria have been established to adjust the GM dose to less than one µg/dL [30]. It is essential to induce negative pressure with iSAP or iJAP tubes, as it has been confirmed [17] that without negative pressure induction when administered from the bone marrow needle, the procedure would simply become a bone marrow infusion, and the drug would be perfused from the bone marrow into the venous return. This possibility cannot be ruled out. After this case, the negative pressure induction technique was ensured, and no further renal dysfunction occurred.

DAIR can be performed first for PJI without significant loosening, as this CLAP technique allows for precise local perfusion of GM, a concentration-dependent antimicrobial agent, at concentrations above MBEC. However, the number of cases is small (six cases), the average follow-up period is approximately two years, and there are no cases of MRSA [27], which is considered to be one of the factors contributing to poor outcomes in PJI, so it is necessary to further increase the number of cases in the future and to investigate the treatment outcomes of CLAP for various types of late-phase postoperative TKA. We want to increase the number of cases in the future and to accumulate results and study the outcomes of CLAP therapy for various kinds of chronic postoperative TKA infection. Therefore DAIR combined with CLAP can be a good alternative for those patients in whom prosthesis removal cannot be considered.

### Electronic supplementary material

Below is the link to the electronic supplementary material.

**Supplementary Material 1: Fig. A** Dual-lumen tubes (Salam samp tube®: Nihon Covidien Co.)

**Supplementary Material 2: Fig. B** Bone marrow needles (iMAP needle®: Cubex Medical Co.)

**Supplementary Material 3: Fig. C** Appearance of tubes and other equipment during implementation of CLAP therapy.

**Supplementary Material 4: Fig. D** Appearance of four syringe pumps in the implementation of CLAP therapy.

**Supplementary Material 5: Fig. E** Case2 Local findings of both leg at the time of first visit to our hospital Appearance of local findings.

**Supplementary Material 6: Fig. F** Case2 Local findings of both leg at the time of first visit to our hospital Thermography findings.

**Supplementary Material 7: Fig. G** Case2 Simple X-ray image at the time of the first visit to this hospital AP view.

**Supplementary Material 8: Fig. H** Lateral view.

**Supplementary Material 9: Fig. I** Case 2 Intraoerative findings around knee joint.

**Supplementary Material 10: Fig. J** Case 2 Purulent effusions collected from within the knee joint.

**Supplementary Material 11: Fig. K** Case 2 After removal of the articular surface and thorough cleaning and debridement using a pulse washer.

**Supplementary Material 12: Video 1** Confirmation that saline injected through a bone marrow needle inserted into the distal femur is drained from within the knee joint.

**Supplementary Material 13: Video 2** Identification of purulent effusions draining from within the knee joint after TKA.

**Supplementary Material 14: Video 3** Saline injected from a bone marrow needle inserted into the femoral diaphysis is drained from the distal femoral fragile area and intercondylar areas.

**Supplementary Material 15**: Case 2.

**Supplementary Material 16**: Surgical procedure.

## Data Availability

The datasets used and analyzed during the current study are available from the corresponding author upon reasonable request.

## Abbreviations

TKA: Total knee arthroplasty
PJI: Periprosthetic Joint Infection
CLAP: Continuous Local Antibiotic Therapy
iMAP: Intramedullary antibiotics perfusion
iSAP: Intrasoft tissue antibiotic perfusion
iJAP: Intrajoint antibiotic perfusion
DAIR: Debridement, antibiotics, and implant retention
NPWT: Negative Pressure Wound Therapy
GM: Gentamicin
MIC: Minimal inhibitory concentration
MBEC: Minimal biofilm eradication concentration
MRSA: Methicillin-resistant *Staphylococcus aureus*
MSSA: Methicillin-susceptible *Staphylococcus aureus*

## Declarations

### Authors’ information

The authors and Maruo et al. run an organization called the CLAP Study Group (https://www.ismap-clap.com/information), which promotes and raises awareness of CLAP therapy in Japan.
