# Supplementary figures and images for: DAIR in treating chronic PJI after total knee arthroplasty using continuous local antibiotic perfusion therapy: a case series study
Source: BMC Musculoskelet Disord. 2024 Jan 5;25:36. doi: 10.1186/s12891-024-07165-y (PMC10768161; doi:10.1186/s12891-024-07165-y)

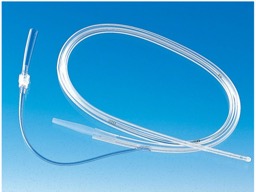

Supplement: Supplementary file 1 — Supplementary Material 1: Fig. A Dual-lumen tubes (Salam samp tube®: Nihon Covidien Co.) [file 12891_2024_7165_MOESM1_ESM.jpg]

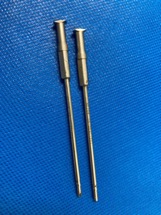

Supplement: Supplementary file 2 — Supplementary Material 2: Fig. B Bone marrow needles (iMAP needle®: Cubex Medical Co.) [file 12891_2024_7165_MOESM2_ESM.jpg]

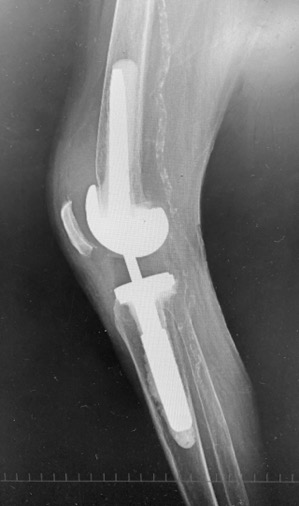

Supplement: Supplementary file 3 — Supplementary Material 3: Fig. C Appearance of tubes and other equipment during implementation of CLAP therapy. [file 12891_2024_7165_MOESM3_ESM.jpg]

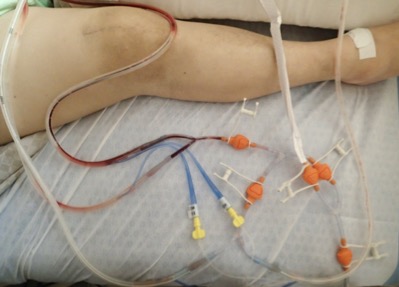

Supplement: Supplementary file 4 — Supplementary Material 4: Fig. D Appearance of four syringe pumps in the implementation of CLAP therapy. [file 12891_2024_7165_MOESM4_ESM.jpg]

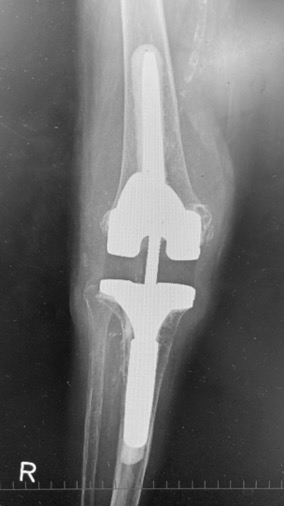

Supplement: Supplementary file 5 — Supplementary Material 5: Fig. E Case2 Local findings of both leg at the time of first visit to our hospital Appearance of local findings. [file 12891_2024_7165_MOESM5_ESM.jpg]

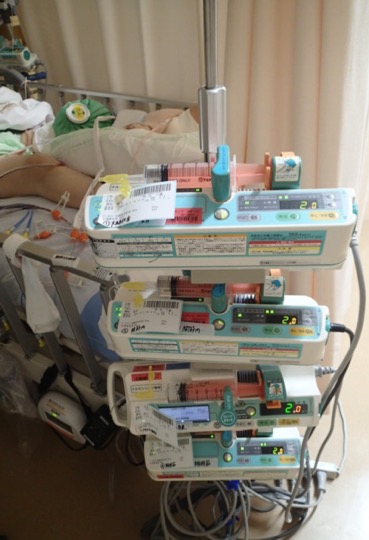

Supplement: Supplementary file 6 — Supplementary Material 6: Fig. F Case2 Local findings of both leg at the time of first visit to our hospital Thermography findings. [file 12891_2024_7165_MOESM6_ESM.jpg]

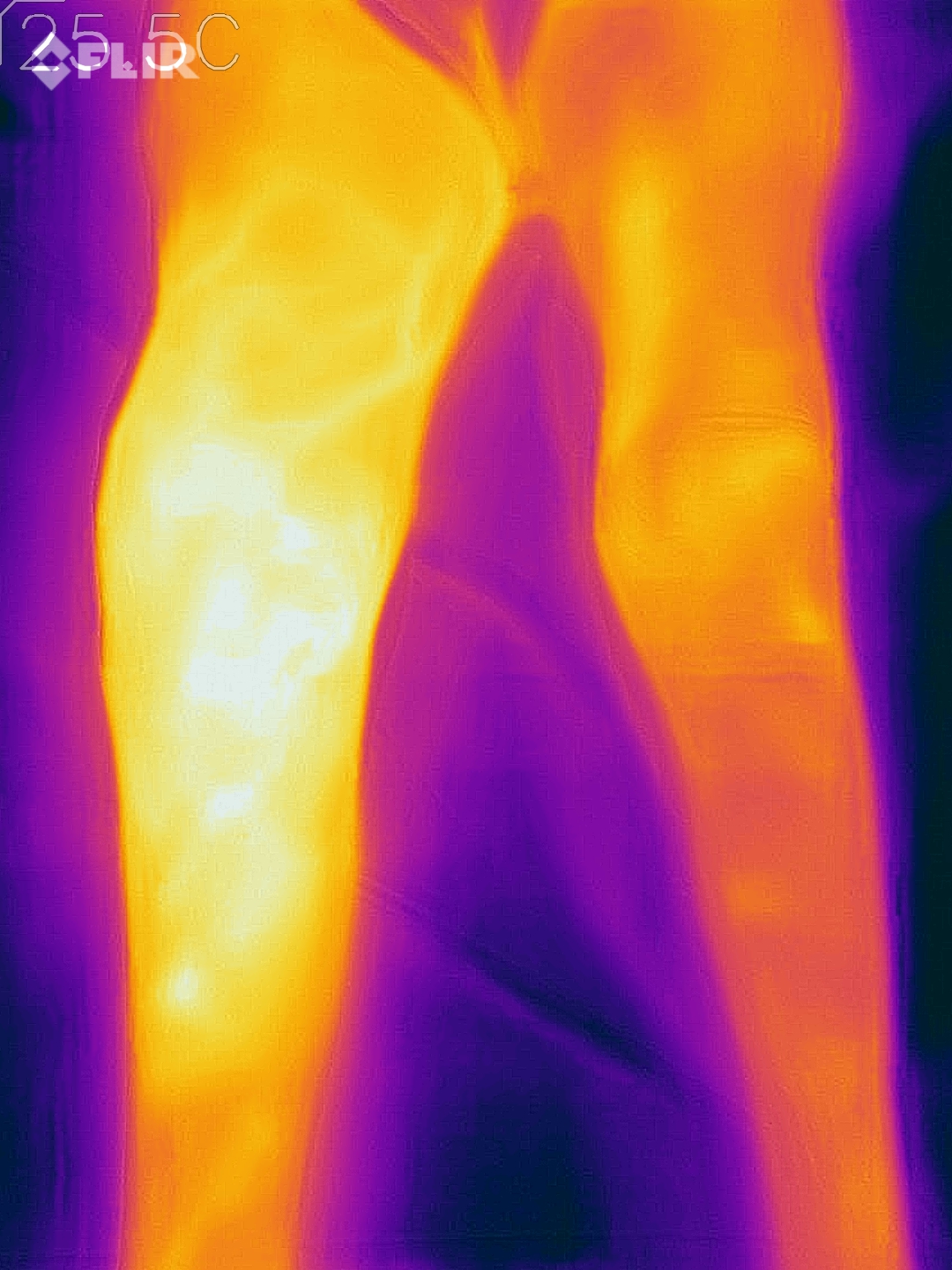

Supplement: Supplementary file 7 — Supplementary Material 7: Fig. G Case2 Simple X-ray image at the time of the first visit to this hospital AP view. [file 12891_2024_7165_MOESM7_ESM.jpg]

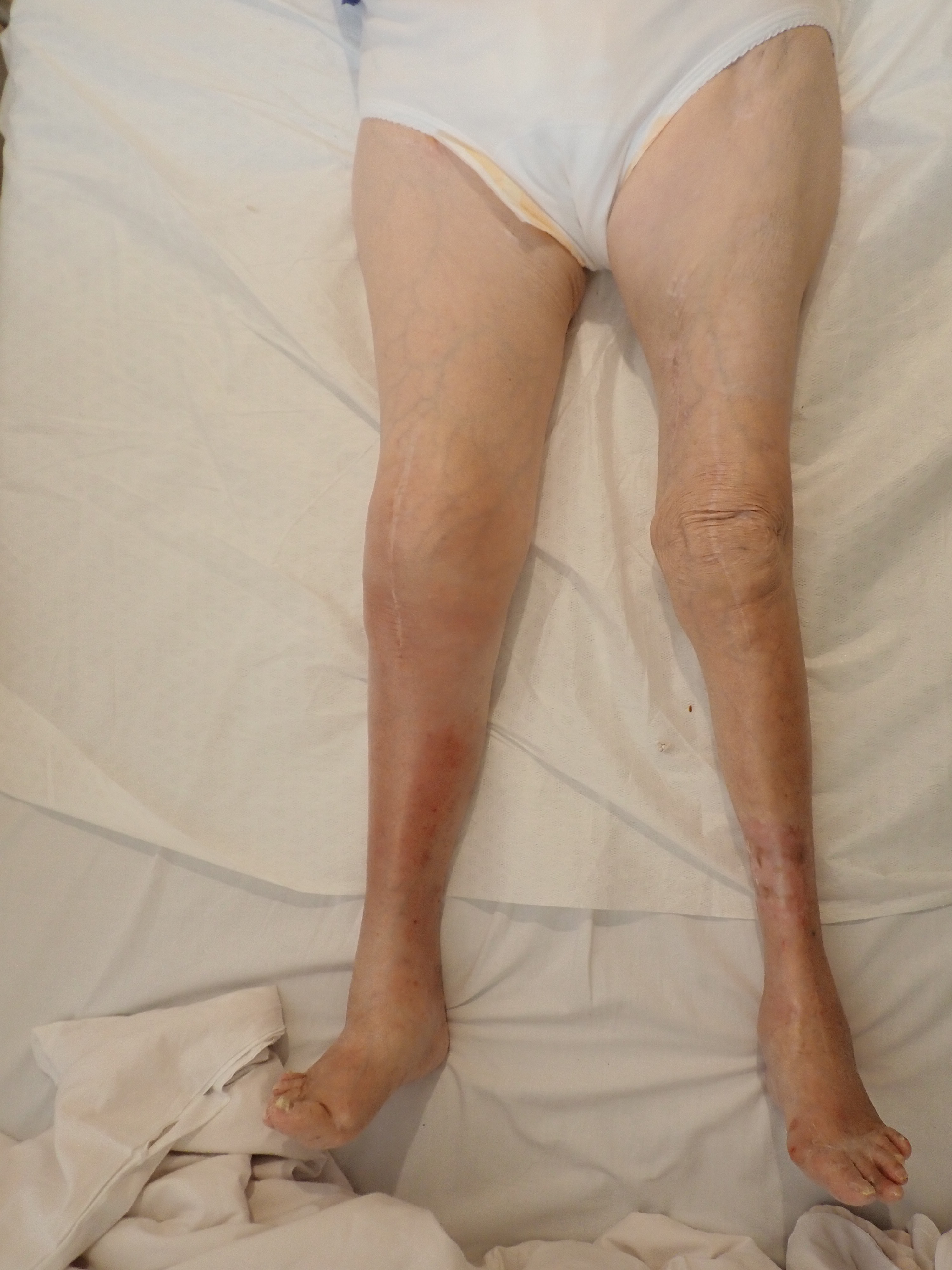

Supplement: Supplementary file 8 — Supplementary Material 8: Fig. H Lateral view. [file 12891_2024_7165_MOESM8_ESM.jpg]

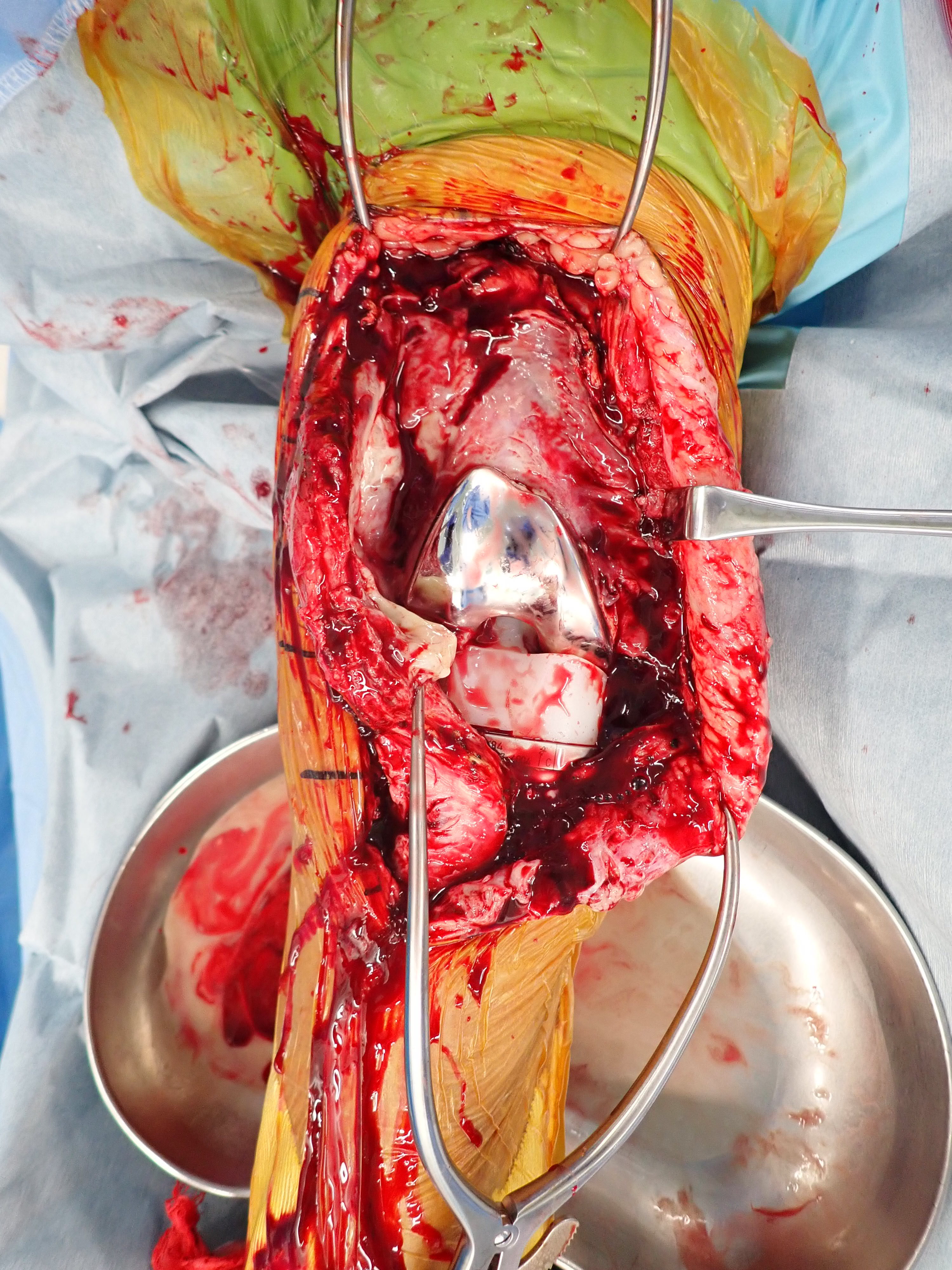

Supplement: Supplementary file 9 — Supplementary Material 9: Fig. I Case 2 Intraoerative findings around knee joint. [file 12891_2024_7165_MOESM9_ESM.jpg]

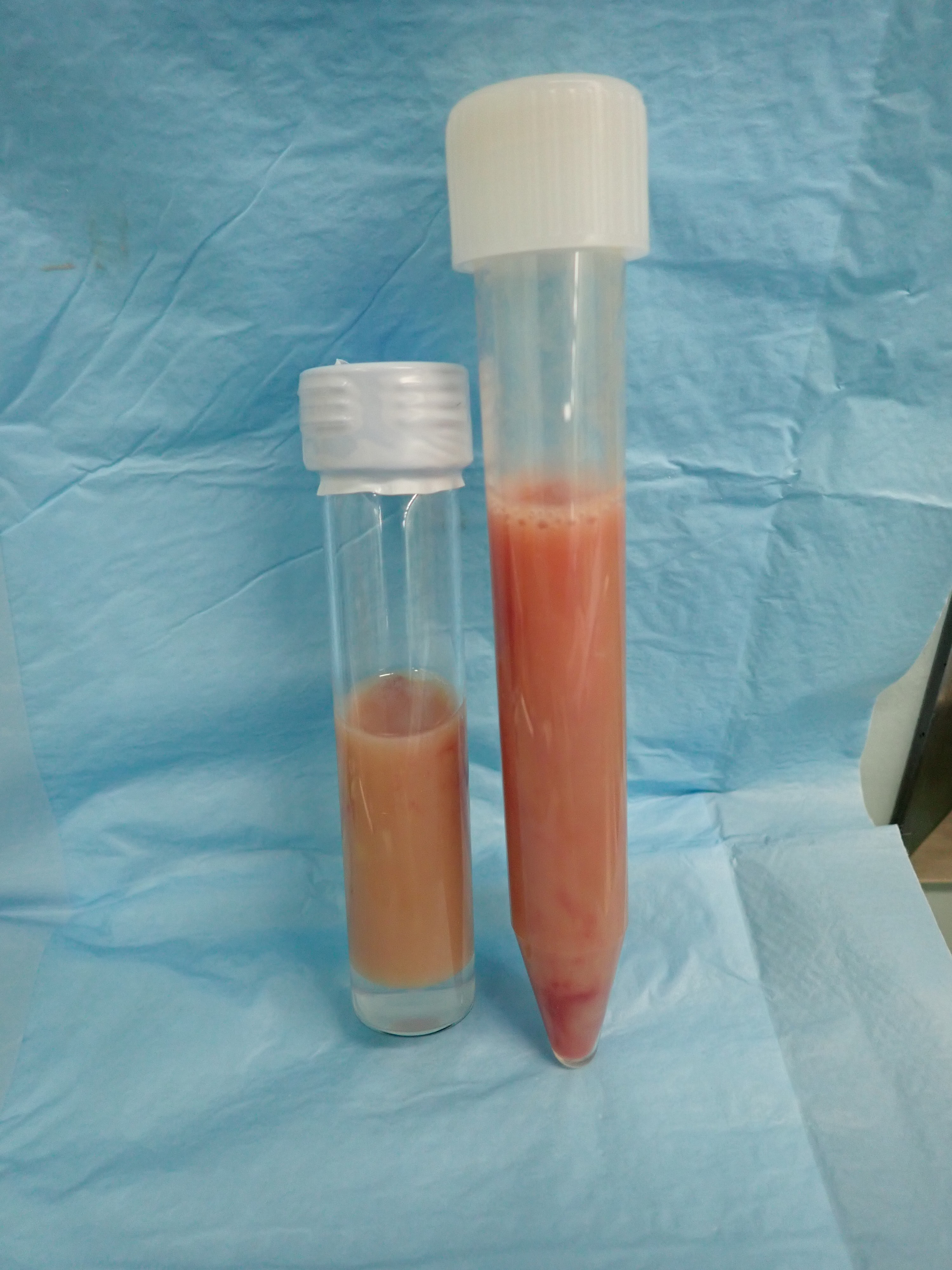

Supplement: Supplementary file 10 — Supplementary Material 10: Fig. J Case 2 Purulent effusions collected from within the knee joint. [file 12891_2024_7165_MOESM10_ESM.jpg]

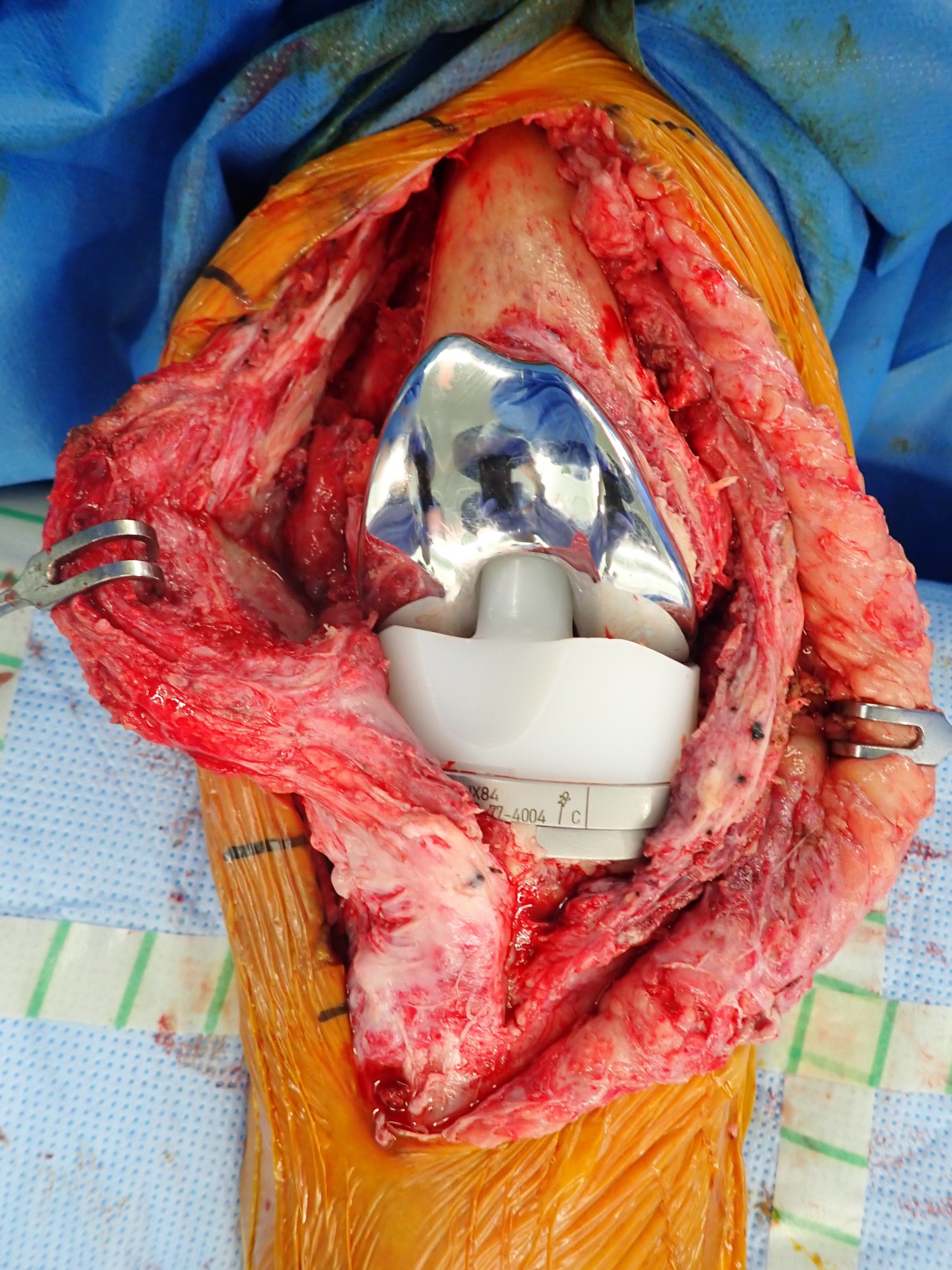

Supplement: Supplementary file 11 — Supplementary Material 11: Fig. K Case 2 After removal of the articular surface and thorough cleaning and debridement using a pulse washer. [file 12891_2024_7165_MOESM11_ESM.jpg]
