# Supplementary material for: DAIR in treating chronic PJI after total knee arthroplasty using continuous local antibiotic perfusion therapy: a case series study
Source: BMC Musculoskelet Disord. 2024 Jan 5;25:36. doi: 10.1186/s12891-024-07165-y (PMC10768161; doi:10.1186/s12891-024-07165-y)

***Case 2***

*Patient*

A 75-year-old woman, medical history: rheumatoid arthritis (biologics user).

*Current medical history*

The patient had undergone revision TKA at this hospital seven years and nine months earlier and had no major postoperative problems, but without any specific trigger, swelling of the right knee joint appeared, and pain was observed, which led to an emergency visit to this hospital.

*Local findings*

Swelling, heat, and redness of the right knee joint (Fig. E), and both automatic and voluntary movements in the same area were difficult due to pain. Thermographic comparison of the affected and healthy sides clearly showed hot lesions on the affected side, mainly in the right knee joint (Fig. F).

*Imaging findings*

A simple X-ray of the left knee joint at the first visit to the clinic showed no obvious loosening of the implant (Fig. G and H).

*Blood data*

Blood test results at the first visit to our department were WBC 12.8, RBC 4.06, HGB 9.7, PLT 217, Neutro 83.5%, TP 6.3, Alb 3.5, CK 74, and CRP 3.34.

*Intraoperative findings*

The case was deployed using a previous skin incision (lateral parapatellar approach), and a marked purulent effusion was found when the inside of the joint was deployed (Fig. I). The effusion and the faulty intra-articular tissue were submitted to bacterial culture (Fig. J), but no inflammatory organisms were identified. iJAP was performed after removal of the articular surface and thorough cleaning and debridement with a pulse washer (Fig. K). iJAP was performed by placing three tubes in the joint (lateral, medial, and intercondylar regions), and CLAP therapy was performed.

*Postoperative course*

Immediately after the operation, the swelling around the right knee joint was quickly reduced, and the pain was alleviated. The iJAP tube was removed on Day 17, as the patient was doing well without any adverse events. One year and six months after surgery, there was no recurrence of infection and no obvious loosening of the implant on a simple X-ray image, and the patient was living her daily life without any particular problems.

Fig.E Fig.F Fig. G Fig. H


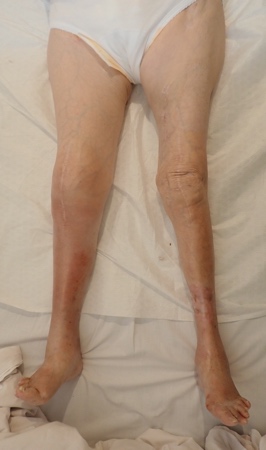

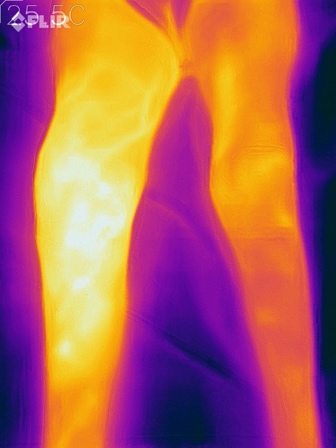

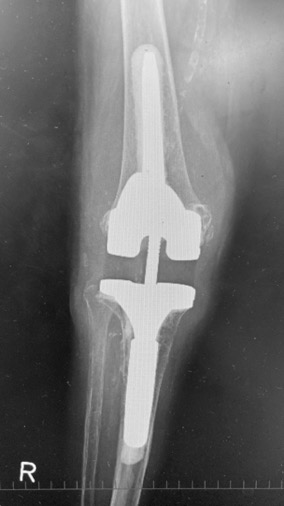

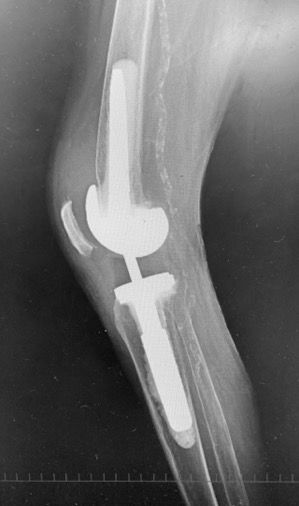


Fig. I Fig. J Fig. K


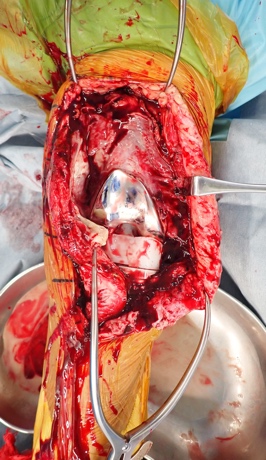

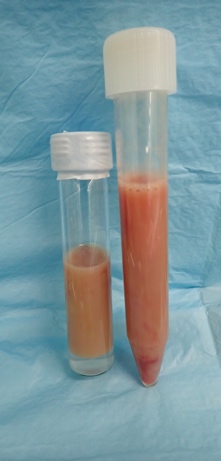

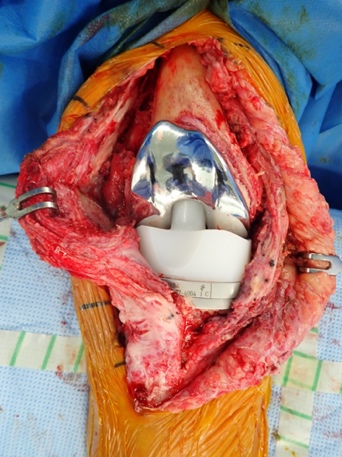

Supplement: Supplementary file 15 — Supplementary Material 15: Case 2. [file 12891_2024_7165_MOESM15_ESM.docx]
