# Supplementary material for: DAIR in treating chronic PJI after total knee arthroplasty using continuous local antibiotic perfusion therapy: a case series study
Source: BMC Musculoskelet Disord. 2024 Jan 5;25:36. doi: 10.1186/s12891-024-07165-y (PMC10768161; doi:10.1186/s12891-024-07165-y)

***Surgical procedure***

The operation was developed from the previous skin incision (lateral parapatellar approach), and the patella was retracted medially while exposing the inside of the joint. Intra-articular effusions and insufficient tissue were collected for submission to bacterial culture tests, after which intravenous antimicrobials were started. Washing and debridement were performed to thoroughly remove the defective tissue within the joint. Washing involved applying at least 10 liters of saline with a pulse washer. A small amount of povidone-iodine and oxide:hydrogen peroxide solution was mixed as appropriate. The joint surface was changed in all cases and sizes, while joint instability was assessed intraoperatively. The most important aspect of this procedure was identifying the infection’s primary site. In cases where the preoperative CT images suggested an infected lesion in the bone marrow or the intraoperative findings, e.g., fragility in the cortical bone or intercondylar area, suggested that infection may have spread into the bone marrow, the bone marrow was also thoroughly cleaned using a dedicated bone marrow needle(Video1). The placement of the tube as CLAP therapy was on a case-by-case basis. Two or three double-lumen tubes were placed in the joint along with a bone marrow needle if the infection had spread into the bone marrow. However, creating a bony foramen in the cortical bone or articular surface was essential to allow irrigation subcutaneously or within the joint so that fluid from the bone marrow needle placed in the bone marrow did not become a bone marrow drip. Double-lumen tubes placed subcutaneously or within the joint could be negatively pressurized to perform infusion and drainage collection by negative pressure loading simultaneously. Negative pressure was applied continuously at 40-80 mmHg using a localized negative pressure wound therapy: NPWT (RENASYS^®️^; Smith & Nephew Co.) device. In addition, drug infusion into the affected area was performed by connecting one tube or bone marrow needle to a syringe pump (Fig. C and D) with GM 60 mg + 50 ml of saline and continuous infusion at 2 ml/hr. Once a day, 20 mg GM + 20 ml of saline was flushed from the indwelling line during a drug change in the syringe pump, and this was performed to prevent occlusion and diffusion subcutaneously, within the joint, and into the bone marrow lines.

Fig. C Fig. D


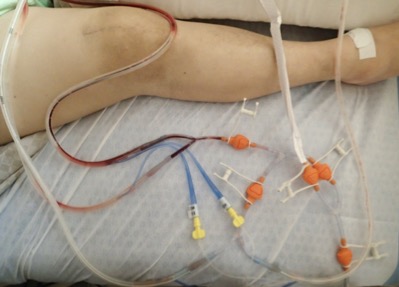

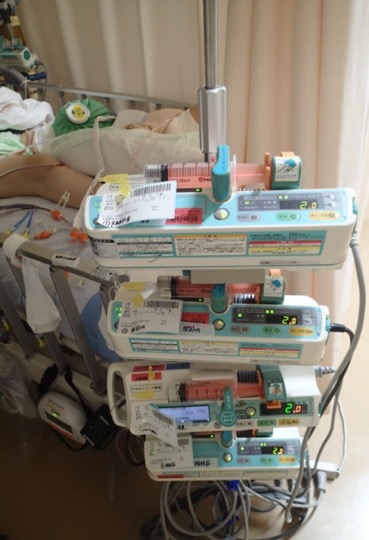

Supplement: Supplementary file 16 — Supplementary Material 16: Surgical procedure. [file 12891_2024_7165_MOESM16_ESM.docx]
